# Real‐life research projects improve student engagement and provide reliable data for academics

**DOI:** 10.1002/ece3.9593

**Published:** 2022-12-08

**Authors:** Sarah A. Marley, Alessandro Siani, Stuart Sims

**Affiliations:** ^1^ Scotland's Rural College (SRUC) Aberdeen UK; ^2^ School of Biological Sciences University of Portsmouth Portsmouth UK; ^3^ Academic & Learning Enhancement, Information & Library Services University of Greenwich London UK

**Keywords:** higher education, practical work, real‐life research projects, student engagement

## Abstract

Student engagement can have a positive influence on student success. Many methods exist for fostering engagement but tend to be generic and require tailoring to specific contexts, subjects, and students. In the case of undergraduate science students, practical classes are a popular tool for increasing engagement. However, despite strong potential for improvement via links with “real life” research projects (RLRPs), few academic staff incorporate research participation with teaching activities. This is potentially due to poor time availability and low opinions of students' ability to collect reliable data. This study aims to examine whether involvement with RLRPs can generate reliable scientific data and also act as a motivational tool for engaging tertiary science students. A preexisting core activity for first‐year biology and marine biology students was modified to include a short RLRP component. Student‐based data collection and a questionnaire about experiences were used to examine the reliability of student‐collected data and student perceptions of RLRPs. Results indicated that error rate in student‐collected data was minimal. Irrespective of participating in a “normal” practical class or a class with a RLRP component, students collected equally accurate data. However, when the topic aligned specifically with their degree subject, student accuracy was higher. All students surveyed reported high motivation with the idea of RLRP participation, placing high importance on this from an educational and employability perspective. Yet, students were not confident about participating in RLRPs until they had engaged with one, suggesting that introducing such projects into taught sessions early‐on may encourage students to seek further opportunities in the future. In conclusion, incorporating RLRPs into the curriculum of undergraduate science courses has considerable potential benefits for both students and academic staff.

## Graphical abstract

Few academic staff involve undergraduate students in research activities, possibly due to low opinions of students' ability to collect reliable data. This study demonstrated that the error rate in student‐collected data was minimal and that students found the idea of participating in research projects highly motivating. Merging research and teaching activities has considerable potential benefits for both students and academics.

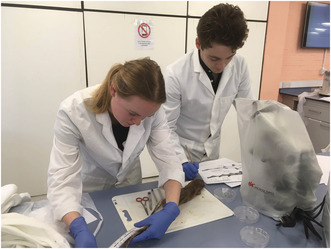

## 1 INTRODUCTION

Student success can be defined in many ways and influenced by a range of factors. Although numerous studies focus on academic achievement, success can also involve acquisition of general knowledge; development of competence, cognitive skills, and intellectual dispositions; preparation for adulthood and citizenship; and personal development (Braxton, 2006). Therefore, when considering student success, it is important to look beyond grades or degree attainment and also consider acquisition of desired knowledge, transferable skills, and competencies (Kuh et al., 2007).

Regardless of how student success is defined, some of the key contributors are background characteristics such as ethnicity, family income, and first‐generation status (Lundberg et al., 2007; Powell et al., 1990; Smith & White, 2015). These characteristics are beyond the control of educators; however, an additional aspect that contributes to student success is engagement. Student engagement is both an intrinsic and an extrinsic factor; it reflects the quality of effort students devote to educationally purposeful activities and the effort institutions devote to using effective educational practices (Kuh, 2001; Kuh et al., 2007). It can also be considered as both an outcome and a process; as students become engaged, their involvement can promote ever greater engagement (Reschly & Christenson, 2012). Indeed, engagement can potentially gain a metacognitive aspect as students become aware of their own learning process (Haave, 2016; Hacker, 1998; Larmar & Lodge, 2014; Marra et al., 2021; Tanner, 2012). Engagement is important because it is an aspect within the control of educators that can positively influence student success. For example, engagement is positively related to academic grades, critical thinking, and persistence among tertiary students (Carini et al., 2006; Finn & Zimmer, 2012; Fraysier et al., 2020; Kuh et al., 2008; McCormick et al., 2015; Schudde, 2019).

There are a number of existing proposals for fostering student engagement, which are typically categorized according to a four‐strand conceptual organizer (Zepke & Leach, 2010): (1) Motivation and agency, where engaged students are intrinsically motivated and want to exercise their agency; (2) transactional engagement, where students and teachers engage with each other; (3) institutional support, where institutions provide an environment conducive to learning; and (4) active citizenship, where students and institutions work together to enable challenges to social beliefs and practices. Institutional support and active citizenship are typically controlled at the institution level, whereas motivation and agency and transactional engagement can be more flexible as they are typically controlled at the educator level. Therefore, motivation, agency, and transactional engagement arguably offer some of the greatest opportunities for educators to foster student engagement.

### 1.1 Student motivation, agency, and transactional engagement

Motivation is of particular pedagogical interest, although the relationship between motivation and student success is complex. This is partly because motivation is defined as an “internal force that determines the goals of a person” (Sutherland, 1995). It is therefore a hypothetical construct that is difficult to test; consequently, it is often inferred from behavior (Breen & Lindsay, 1999). Furthermore, learning motivation can be intrinsic (e.g., striving to achieve understanding) or extrinsic (e.g., striving to obtain high grades) (Areepattamannil et al., 2011). Typically, engaged students are intrinsically motivated (Zepke & Leach, 2010), and thus invest more effort and take greater care in their work. This is rewarded by higher student achievement and success in comparison with extrinsically motivated students (Areepattamannil et al., 2011). In some cases, strong motivation can even offset student background characteristics to positively influence student success through heightened engagement (Allen, 1999).

Similarly, agency (i.e., control over one's learning activities) can also improve engagement. Agency allows students the opportunity to learn how to make decisions to successfully complete tasks, but it also fosters the motivation to persevere in the face of difficulties (Vaughn, 2020). When students are required to take responsibility for activities, they become invested in the activity and more committed to their studies (Kuh et al., 2008). Agentic engagement has been shown to improve students' achievements and mitigate disengagement (Anderson et al., 2019; Reeve & Tseng, 2011). Beyond individual outcomes, increased student agency can also facilitate broader societal outcomes, such as improved intercultural relationships, internationalism, and globalism (Stenalt & Lassesen, 2022). A key feature of agency is that it allows learning experiences to move beyond a transactional approach, fostering collaboration between students and educators (Vaughn, 2020).

Positive interactions between students and educators are central to successful engagement (Kuh et al., 2006). Approachable teachers who create inviting learning environments are available to discuss student performance and offer student support are more likely to experience heightened student engagement, performance, retention, and loyalty (Bryson & Hand, 2007; Mearns et al., 2007; Snijders et al., 2020). Positive relationships with staff can also promote a sense of belonging within students, particularly those from ethnic minorities (Meeuwisse et al., 2010). Students themselves also recognize that strong relationships with staff are important (Snijders et al., 2018). It is therefore important to not only create chances for student–teacher interactions, but also allow opportunities for these to develop into quality relationships. Therefore, it is important for educators to design activities that promote engagement, intrinsic motivation, learner responsibility, and interaction between students and staff.

Proposals for improving student engagement that are linked to motivation, agency, and transactional engagement include the following: enhancing students' self‐belief; enabling students to work autonomously; recognizing that teaching and teachers are central to engagement; creating active‐learning environments; fostering collaborative learning; and generating challenging educational experiences (Russell & Slater, 2011; Zepke, 2013; Zepke & Leach, 2010). However, implementing these generic proposals can be challenging for educators. There is a need to investigate how these suggestions can be successfully integrated for specific subjects and students.

### 1.2 Engagement via practical classes

In the case of undergraduate science students, a teaching method that has been shown to increase student engagement is the use of practical classes (Charney et al., 2007). Practicals can take a range of forms, from teacher‐led demonstrations to “recipe‐style” activities to independent research projects (Dunlop et al., 2019). These activities offer an opportunity to develop conceptual knowledge, technical skills, and general scientific literacy (Areepattamannil et al., 2011; Ferreira & Morais, 2020; Freedman, 1997; Hofstein & Lunetta, 2004; Millar & Abrahams, 2009; Tobin, 1990). Within practical classes, exercises commonly involve cooperative learning in groups, which also enhances competencies related to social interaction (Goldschmidt & Bogner, 2016). Students themselves recognize the importance of such classes, ranking “technical skills” of high importance and second only to learning the general theory of their subject (Edmondston et al., 2010). They also find practicals useful and enjoyable compared with other science teaching and learning activities, with student enjoyment strongly linked to better learning (Abrahams & Millar, 2008; Kickert et al., 2022). Practicals have positive links with the aforementioned concepts of student engagement. Class sizes are typically smaller than those of lectures, allowing more frequent and higher quality transactional engagement between students and educators. Furthermore, practicals allow development of student motivation and the opportunity to exercise agency. Consequently, practical classes have a distinctive and central role in science curricula (Goldschmidt & Bogner, 2016).

However, given the logistical complexities involved in planning practicals, these activities are at risk of being ineffective (Millar & Abrahams, 2009). Without a clear and precise purpose, activities can be ill‐conceived, confused, poorly explained, and unproductive (Hodson, 1991; Kulgemeyer, 2018). In such cases, practical work fails to foster scientific thinking skills and cognitive achievement (Abrahams & Millar, 2008). Practical activities also risk being overly prescriptive; this constrained agency can lead to dissatisfaction and underperformance (Stenalt & Lassesen, 2022). Students are quick to perceive activities as “meaningless” and thus attribute them to having low value (Abrahams & Reiss, 2012), which is a particular risk of nonassessed activities (Kickert et al., 2022). This perception of meaningfulness and value is important for student motivation; when students perceive value in tasks, they will actively engage and use active‐learning strategies, whereas when students do not perceive value, they use surface‐learning strategies (Tuan et al., 2005). Thus, there is a need to carefully plan practical classes to maximize their positive impacts and ensure such activities are valuable for students.

### 1.3 Engagement via “real life” research projects (RLRPs)

One method for strengthening the value and benefits of practical classes is by creating links with academic research. Although many undergraduate science students complete a course‐based undergraduate research project in their final year, there is benefit to engaging students with research earlier in their degrees in order to facilitate progressive capacity development throughout the entire course (Brew, 2013; Clark & Hordosy, 2019; Howell, 2021). Staff research activity positively influences student learning (Guerin & Ranasinghe, 2010; Guo et al., 2018; Hattie & Marsh, 1996; Jenkins et al., 1998; Neumann, 1992; Ramsden & Moses, 1992), and involvement in academic‐led “real life” research projects (RLRPs) facilitates student‐centered learning in an authentic learning environment (Breen & Lindsay, 1999; Charney et al., 2007; Healey et al., 2010). The student is immersed in a collaborative environment where practical skills are connected to real science through meaningful tasks (Bigot‐Cormier & Berenguer, 2017; Charney et al., 2007). Involvement in such projects has the potential to be motivating, provide opportunities for agency, and allow collaborations between students and researchers. Students thus perceive involvement in research as valuable, and even enjoyable. Indeed, previous studies have found an association between higher course satisfaction and students with positive attitudes toward research, which included students having an interest in research and wishing to participate in research (Breen & Lindsay, 1999; Healey et al., 2010; Howell, 2021; Jenkins et al., 1998). Involvement in RLRPs has also been reported to positively influence the future career decisions of students (Dunlop et al., 2019).

Yet, few tertiary lecturers appear to incorporate research participation with teaching activities. This may be the result of poor time availability to design and trial such activities, a conflict in time between teaching and research, or low opinions of students' ability to collect accurate and reliable data and positively contribute to research outcomes (Brew & Mantai, 2017; Hattie & Marsh, 1996; Kloser et al., 2011). Many of the challenges around engaging inexperienced students in research and data collection are shared by the citizen science movement. For example, data quality is widely seen as a problem for those working in citizen science, with a range of strategies undertaken to a ameliorate this, including close supervision, cross‐checking results, and simplifying tasks (Riesch & Potter, 2014). Indeed, Mitchell et al. (2017) discovered that engaging undergraduate students in citizen science actually decreased the students' own perception about the accuracy of measurements taken and the usefulness of such data, although engagement increased. However, while a wealth of data is collected annually as part of Course‐Based Undergraduate Research Experiences (CUREs), these observations very rarely go beyond the classroom, irrespective of their quality or originality (Messager et al., 2022). Conversely, involving students in RLRPs allows them to participate in projects that have real‐world applications, proving the usefulness of research in general and their own skills in particular. Involving students in the research of teaching staff can also could be more time‐efficient for academics by providing additional opportunities for research and a higher number of “person hours” for data collection (Harland, 2016; Tight, 2016). Yet, many academic staff remain to be convinced of the quality of student‐collected data, to the detriment of their own research and student learning.

### 1.4 Study aims

This study aims to examine whether involvement with RLRPs can generate reliable scientific data and be used as a motivational tool for engaging tertiary science students. Specifically, this study will (1) measure the overall reliability of student‐collected data in practical classes; (2) compare the accuracy of student‐collected data in classes with and without a RLRP component; and (3) evaluate student perceptions of RLRPs. Findings will first demonstrate whether student‐collected data are of sufficient quality for inclusion in academic research projects, which will be of use to staff considering such data gathering activities. Findings will also indicate whether student motivation increases through involvement in research projects by comparing students participating in classes with and without a RLRP component, both through student surveys exploring perceptions and by comparing error rates of student‐collected data. This dual approach will allow examination of student motivation in terms of the way students think and the way they behave. Overall, these results will inform academics about the benefits of incorporating RLRPs into their activities, from both a research and a teaching perspective.

## 2 METHODS

This study recruited first‐year students studying BSc Marine Biology and BSc Biology at the University of Portsmouth. As part of a core first‐year module designed to train students in a range of essential laboratory techniques, these students undertake a single‐instance practical to gain familiarity with dissection techniques and spotted dogfish (*Scyliorhinus canicula*) morphology. Students work in pairs, receive a specimen for examination, and are asked to complete a workbook regarding anatomical features.

Convenience sampling was conducted to recruit students from this practical to the current study. This utilized the lead author's previous experience with teaching this activity to ensure study design would not impact learning objectives. This study was undertaken in accordance with the University of Portsmouth Ethics Policy (No: ED182005). All participants were informed of the voluntary and anonymous nature of the study, and of their right to withdraw without any negative repercussions on achievement and progression.

### 2.1 Data collection

A visual overview of the data collection methodology is provided in Appendix A. Due to the size of the first‐year student cohort (120 students), this practical class had three repeats over a three‐day period in January 2020. All classes were delivered by the lead author and supported by the same technician and demonstrating assistant. Each morning, dissection kits and specimens were prepared by the technician to allow one station per student pair. The stations were labeled sequentially, so that each dogfish had an individual identification number. Prior to the start of each class, measurements were taken by the lead author and two laboratory assistants for all dogfish (e.g., total length and fin length). This represented a “ground‐truthed” dataset with which to compare student‐collected measurements.

Approximately 40 students were timetabled to attend each class. The first class was timetabled to contain only Marine Biology students, the second class contained students from both degree streams, and the third class only contained Biology students. Therefore, “pure” classes were initially selected as Experimental Groups while mixed class was kept as a no‐treatment Control Group. Although the authors recognize that this does not represent an ideal experimental design, limited institutional resources and timetabling requirements restricted full educator control over this arrangement. This study limitation is further considered in the Discussion. Additionally, there was one case of a student attending the wrong day, resulting in a single mixed pair in one of the Experimental Groups (see Section 3).

Both Control and Experimental Groups had the same taught material to ensure no unfairness in terms of their education and learning outcomes. This included a description of sexual dimorphism (i.e., where two sexes of the same species exhibit different characteristics), which has been shown to exist in dogfish for a range of anatomical features (Filiz & Taskavak, 2006). This information was used to justify why students were recording measurements from their specimens. However, the Experimental Group was also told that their worksheets would be collected at the end of class to contribute to a scientific study investigating dogfish sexual dimorphism (which is indeed being conducted by the lead author); this was the only orchestrated difference between the Control and Experimental Groups. The importance of collecting accurate scientific measurements was emphasized to all students, regardless of their grouping.

A two‐sided worksheet was given to all pairs for in‐class completion and return. Each worksheet asked the pairs to indicate the degree stream they were from (Marine, Biology, or Both if a mixed pair) and the day of the week their class occurred. This information was collected to try and explain any underlying differences between students; for example, differing experiences between degree streams or communication between students on differing days. No additional background characteristics or demographic data were collected due to logistical challenges of ensuring student privacy while also linking such information to ground‐truthed measurement records. Additionally, given the relatively small class sizes, it is unlikely that sufficient sample sizes representative of different demographic groups would have been captured for statistical analysis. The front page of the worksheet was specific to dogfish measurements and group work; it contained a diagram of a dogfish and indicated eight sites along the body where measurements were to be recorded, along with details on the dogfish sex and ID number (Appendix B). The back page was specific to individual student motivation; it contained six Likert‐style questions (one set per student; Table 1) relating to individual perception of practical classes, their confidence in their own technical skills, and their opinions of involving students in RLRPs. In order to provide some context and further interrogate the impact of the experiments, open‐text comments were collected from students on the survey about their perception of undertaking the measurements. As these were a brief, adjunct to the research, they are not intended for rigorous qualitative analysis (LaDonna et al., 2018). Rather, these comments were categorized based upon a descriptive interpretation of their core focus (e.g., “confidence”). Each statement could have multiple foci, for example, if a student talked about gaining confidence but also acknowledged the possible employment benefits of engaging in the activity. These different foci were then quantified to build an understanding of the range of different perceptions across the cohorts.

**TABLE 1:** List of six Likert‐style questions that students were asked to complete at the end of their practical class.

Number	Question
Question 1	I think practical classes support my learning
Question 2	I enjoyed this practical class
Question 3	I am confident in my technical skills
Question 4	Working on ‘real life’ research projects motivates me
Question 5	I do *not* feel confident in participating in ‘real life’ research projects
Question 6	I think it is important that students are involved in ‘real life’ research projects

*Note*: Question 6 was also followed by an open question seeking further explanation.

### 2.2 Data analysis

Data were analyzed in R (vr 4.2.1) using the packages *car* and *dunn.test* (Dinno, 2017; Fox & Weisberg, 2019). A significance level of 0.05 was used for all analyses (excluding those where a Bonferroni correction was applied).

Overall proportion of complete versus incomplete worksheets were compared by Treatment Group using a chi‐square test for associations. Student‐collected measurements were compared with ground‐truthed measurements to give an error rate; this was taken as an indication of accuracy. Data screening was undertaken using Shapiro–Wilk and Levene's tests; however, data were neither normal nor homogenous. Therefore, nonparametric tests were used to compare the error rate according to three explanatory variables (Table 2). Wilcoxon tests were used to compare the median error rate according to Treatment Group. Pair Composition and Measurement Site were investigated using Kruskal–Wallis tests and post hoc Dunn's tests with Bonferroni corrections.

**TABLE 2:** Definitions of the three explanatory variables used during data analysis

Variable	Levels	Description
Treatment Group	Control Experimental	“Control” students were *not* asked to contribute to a RLRP, whereas “Experimental” students were asked to contribute their measurement data to a RLRP. Included as the key differentiating factor in the study.
Pair Composition	Marine Biology Biology Both	Indicated the degree type being studied by the student pair. Either both students were studying the same degree type or there was one student from each of the two degree streams. Included due to a mix of “pure” classes and mixed classes, and to control for the fact that students did not necessarily stick to their timetabled class.
Measurement Site	1–8	Discrete measurement sites on the dogfish body (see Appendix B). Included to investigate whether student accuracy varied due to complexity of the measurement required (e.g., body size being relatively simpler and more intuitive to measure than mouth size)

Student surveys were analyzed in two parts, both of which utilized chi‐square tests as these are appropriate for both nominal and ordinal data (Kraska‐Miller, 2013; Sirkin, 2006). For each of the Likert‐style questions, chi‐square tests for associations were used to investigate the proportion of students who responded from 1 (Strongly Disagree) to 5 (Strongly Agree) in relation to Treatment Group (Table 2). The open question was manually reviewed to establish key themes in student responses, the occurrence of which were analyzed using chi‐square tests for association in relation to Treatment Group (Table 2).

## 3 RESULTS

### 3.1 Student‐collected data

A total of 53 worksheets were submitted. This included a total of 20 worksheets from the Control Group, with seven Marine Biology pairs, four Biology pairs, and nine pairs from Both. The Experimental Group had a total of 33 worksheets, comprised of 16 Marine Biology pairs, 16 Biology pairs, and one pair from Both.

The majority (81.1%; *n* = 43) of worksheets were fully completed. Of the 10 worksheets that contained omissions, four did not indicate the dogfish sex and six were missing a morphometric measurement; only one worksheet had multiple omissions in the form of two missing measurements. The Control Group had the largest proportion of worksheets with omissions (25.0%; *n* = 5), compared with 15.2% (*n* = 5) of the Experimental Group; however, this did not represent a significant difference between Treatment Groups (*χ* ^2^ = 0.789, *p* = .3744). Omitted measurements were not included in the dataset; however, the remaining measurements from that worksheet were retained for further analysis.

#### 3.1.1 Dogfish sex

Of the 48 worksheets that recorded dogfish sex, the majority (97.9%; *n* = 47) correctly sexed the animals. The one erroneous record occurred in the Control Group, where a female dogfish was mistakenly identified as a male.

#### 3.1.2 Dogfish morphometrics

**FIGURE 1:**
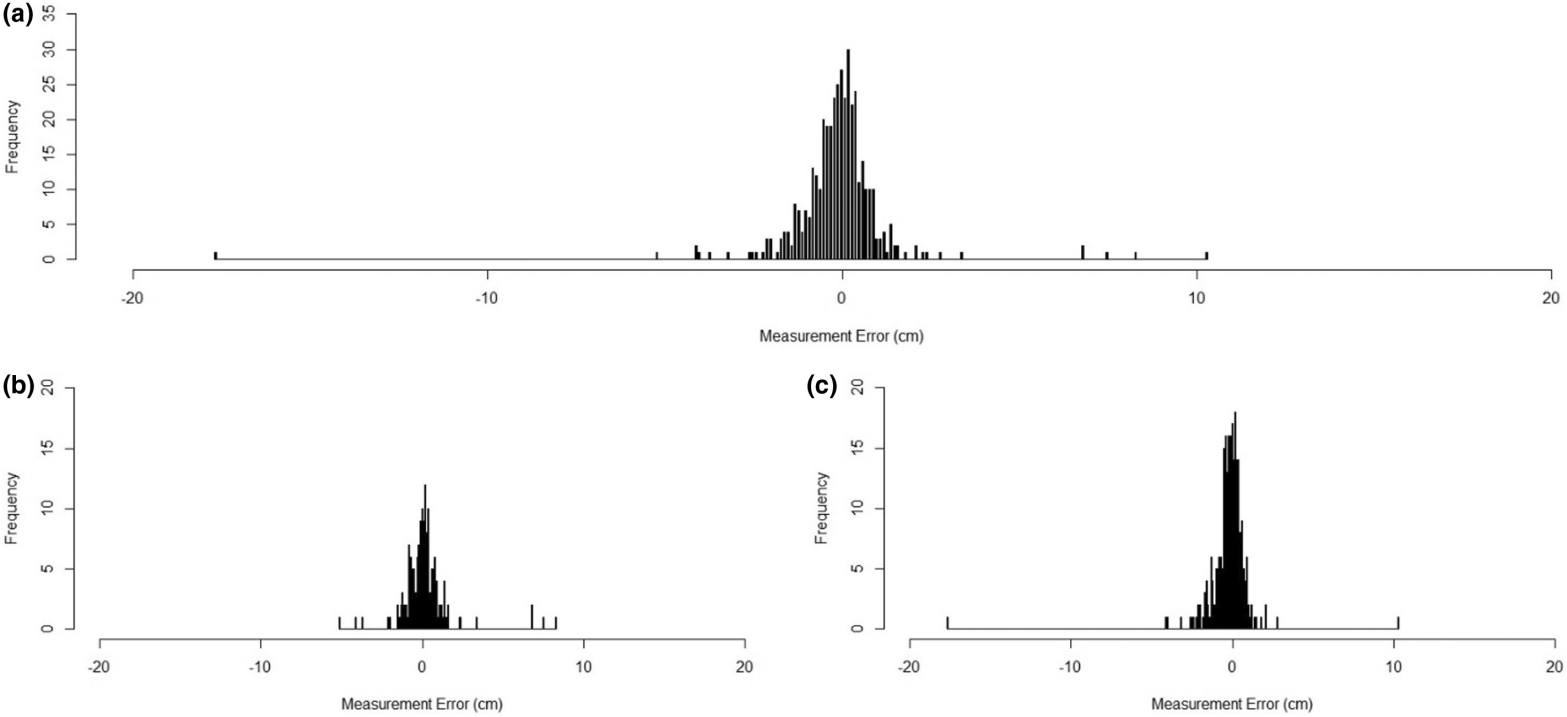
Histogram of measurement errors for (a) all worksheets overall, (b) the Control Group, and (c) the Experimental Group. All data displayed in 0.1 cm bins

The morphometric measurements submitted via worksheets were compared with the “ground‐truthed” records to obtain an error rate. Of the 417 measurements reported, the majority (78.7%; *n* = 328) of worksheet measurements were within ±1 cm of the ground‐truthed records; 50.8% (*n* = 212) were within ±0.5 cm; and 11.8% (*n* = 49) were within the minimum difference of ±0.1 cm (Figure 1a). The maximum error was −17.7 cm; however, this appeared to be the result of the students measuring the dogfish in sections and then incorrectly summing the multiple lengths. When considered by Treatment Group, 76.6% (*n* = 121) of the Control and 79.9% (*n* = 207) of the Experimental measurements were within ±1 cm of the ground‐truthed records; 46.8% (*n* = 74) Control and 53.3% (*n* = 138) Experimental within ±0.5 cm; and 10.8% (*n* = 17) Control and 12.4% (*n* = 32) Experimental within ±0.1 cm (Figure 1b, c).

A Kruskal–Wallis test showed no significant difference in measurement error according to Dogfish ID (*χ* ^2^ = 48.241, df = 52, *p* = .6225); thus, error rates were not specific to particular dogfish specimens or student pairs. However, significant differences did exist according to Treatment Group, Pair Composition, and Measurement Site.

**FIGURE 2:**
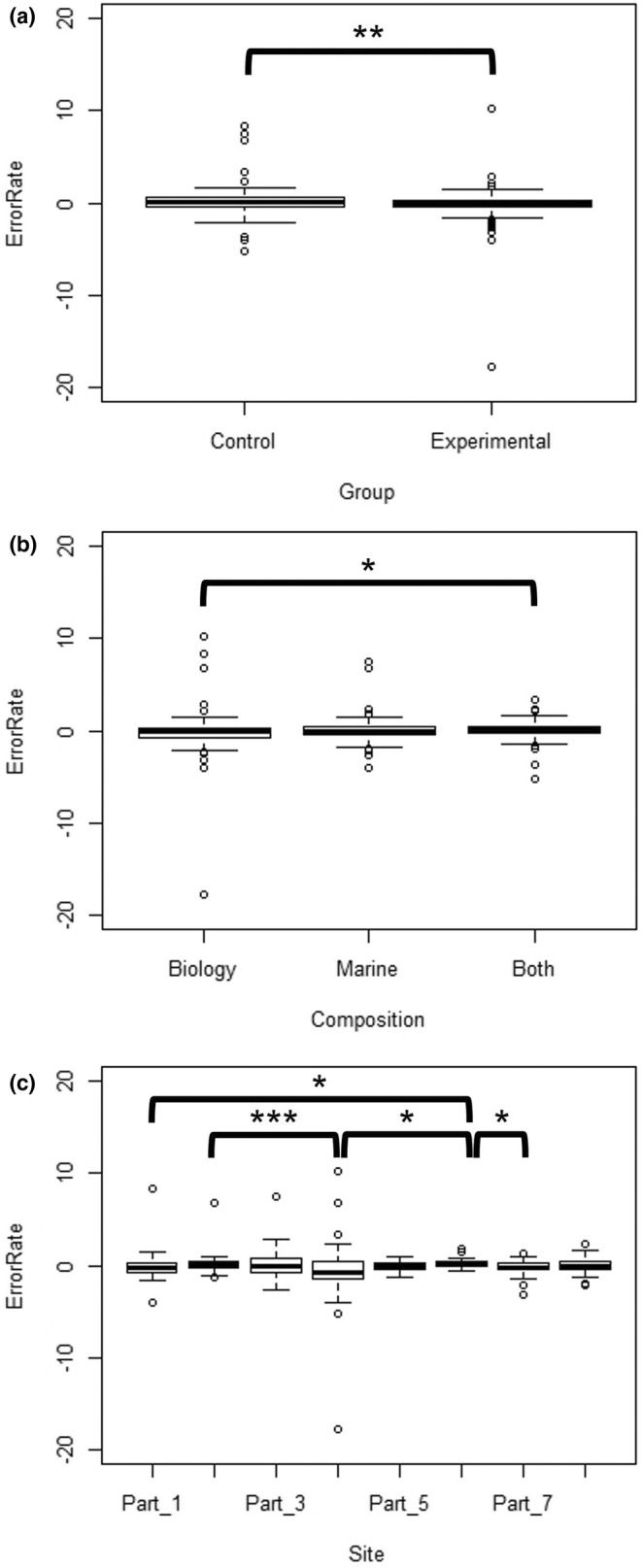
Summary of measurement errors by (a) treatment group, (b) teaching day, (c) pair composition, and (d) dogfish measurement site. Significance level: *** ≤ 0.001; ** ≤ 0.01; * ≤ 0.05

A Wilcoxon Test showed a significant difference in the median error according to Treatment Group (*W* = 23,690, *p* = .007; Figure 2a). The Control Group tended to over‐estimate measurements (median = 0.10 cm), while the Experimental Group under‐estimated (median = −0.10 cm).

A Kruskal–Wallis test indicated a significant difference in the median error according to Pair Composition (*χ* ^2^ = 8.3201, df = 2, *p* = .01561; Figure 2c). A Dunn's test with Bonferroni corrections indicated that pairs mixed from Both degrees were significantly different to Biology‐only pairs (*Z* = −2.6731, *p* = .0113), but there was no significant difference between Biology‐Marine (Z = −2.1253, *p* = .0503) or Marine‐Both (*Z* = 1.0116, *p* = .4676). The median errors were −0.10 cm for Biology, 0.00 cm for Marine, and 0.10 cm for Both.

A Kruskal–Wallis test indicated a significant difference in the median error according to Measurement Site on the dogfish body (*χ* ^2^ = 26.355, df = 7, *p* < .001; Figure 2d). A Dunn's test with Bonferroni corrections indicated that the following parts were significantly different: Parts 6–1 (*Z* = −3.3041, *p* = .0133), Parts 6–4 (*Z* = −4.2790, *p* < .001), Parts 6–7 (*Z* = 3.3474, *p* = .0114), and Parts 2–4 (*Z* = 3.1009, *p* = .0270). The respective median errors for Parts 1 to 8 were − 0.20 cm, 0.10 cm, −0.05 cm, −0.70 cm, −0.05 cm, 0.20 cm, −0.30 cm, and 0.00 cm. Thus, measurements of Part 6 (mouth length) were significantly higher than Part 1, 4, and 7 (body and pectoral fin lengths); additionally, measurements of Part 2 (dorsal fin height) were significantly higher than those of Part 4 (total body length). It is worth noting that both Parts 6 and 2 were challenging areas to measure. Mouth length (Part 6) often had unclear boundaries, while dorsal fins (Part 2) are flimsy body parts that can be manipulated in different ways.

### 3.2 Student surveys

A total of 100 completed survey forms were returned. This included a total of 29 surveys from the Control Group (Pair Compositions: 13 Marine Biology; 5 Biology; 11 Both) and 71 surveys from the Experimental Group (Pair Compositions: 31 Marine Biology; 38 Biology; 2 Both).

#### 3.2.1 Survey questions

**FIGURE 3:**
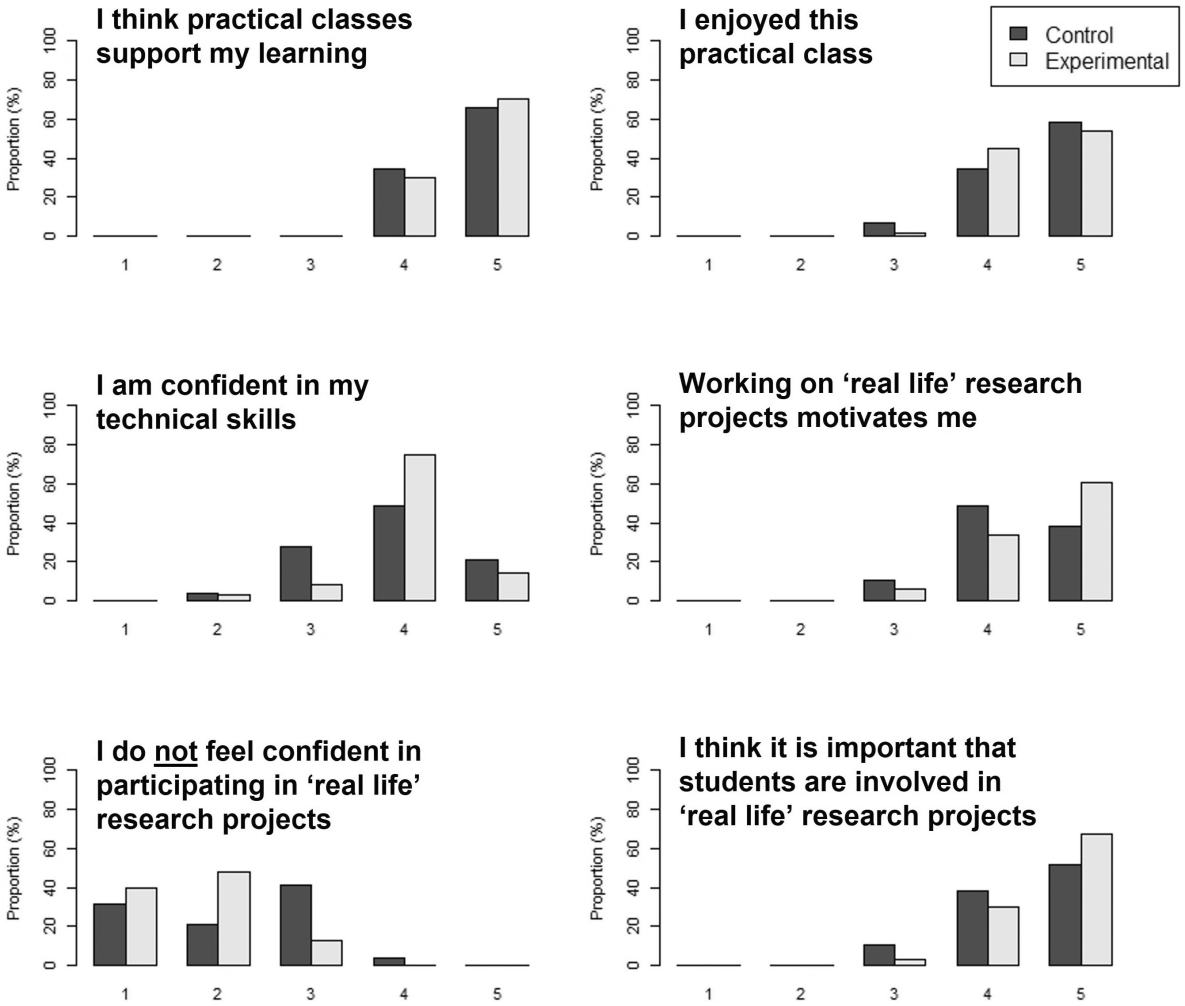
Proportion of responses by treatment group to Likert‐style questions regarding student perceptions of practical classes and ‘real life’ research projects. Responses ranged from 1 (Strongly Disagree) to 5 (Strongly Agree). Significant differences (*p* < .05) were found only in all questions bar Question 1.

The survey responses for all questions are summarized in Figure 3. Overall, students were positive about practical classes supporting their learning (Question 1). They found this particular practical class enjoyable (Question 2), but were variable in regard to their confidence in their own technical skills (Question 3). Students found the idea of working on RLRPs motivating (Question 4) and thought it was important that students were involved in such projects (Question 6), although again had varying levels of confidence in their ability to participate in RLRPs (Question 5).

It was not possible to investigate survey responses by degree stream, as this information not available due to the shared nature of the worksheet; however, responses could be considered by Treatment Group. When the survey responses were considered by Treatment Group, chi‐square tests showed no significant association between Treatment and score for Question 1 (*χ* ^2^ = 0.368, df = 1, *p* = .544). However, there was a significant association between Treatment and score for Question 2 (*χ* ^2^ = 6.253, df = 3, *p* = .044), Question 3 (*χ* ^2^ = 18.438, df = 3, *p* < .001), Question 4 (*χ* ^2^ = 8.612, df = 2, *p* = .013), Question 5 (*χ* ^2^ = 28.928, df = 3, *p* < .001), and Question 6 (*χ* ^2^ = 6.839, df = 2, *p* = .033) (Figure 3). In particular, the Experimental Group showed stronger motivation and greater confidence from working on RLRPs, and also more strongly agreed that it was important for students to be involved with such projects. However, despite being less confident in their ability to participate in RLRPs, the Control Group appeared to enjoy this particular practical class more than the Experimental Group.

#### 3.2.2 Survey themes

A total of 66 students provided 90 answers to the open question, the majority (97.8%; *n* = 88) of the responses spoke positively of student involvement in RLRPs. Only two responses indicating negative opinions, which both indicated that RLRPs could be useful but that students should not be required or forced into participating in such projects.

Of the 88 positive written comments, seven key themes emerged: Experience; Confidence; Comprehension; Cost Effectiveness; Skills; Careers; and Engagement (see Table 3 for examples). Chi‐square tests found no significant association between theme and Treatment Group (*χ* ^2^ = 10.201, df = 7, *p* = .177). It was not possible to investigate the influence of degree stream on themes, as these data were not available at the individual level.

**TABLE 3:** Examples of student responses to the open question asking why it is important for students to be involved in real‐life research projects, which were then categorized into seven key themes.

Theme	Example Comments
Experience (26.1%)	“It gives an insight in how actual research works” “Helps us gain actual experience making it easier when we need to do it”
Comprehension (22.7%)	“Learning is more memorable in practical situations” “Brings us more knowledge than just books or normal classes.”
Career (20.5%)	“It gives students some inspiration for future career and conservation choices” “Employers look for practical skills”
Engaged (10.2%)	“Practical environments help with enthusiasm”^^“It makes time at uni feel well used”
Skills (10.2%)	“Practical classes allow me to apply skills I have learnt as well as improve them by working in groups and learning from each other” “Learn how to do research”
Confidence (6.8%)	“I believe it is important to learn about and be involved in current ‘real life’ research projects as it gives confidence in being a ‘real life’ scientist” “Gives me more confidence”
Cost Effective (3.4%)	“Because science is a collective effort it lightens the load for research and helps the students” “Saves money on research if students are carrying it out”

*Note*: The percentage under each theme name reflect the proportion of written comments that aligned with that theme.

However, chi‐square tests did indicate a significant difference by theme alone (*χ* ^2^ = 42.182, df = 7, *p* < .01; Table 3). The most popular themes reflected students remarking that participating in RLRPs allowed them relevant work and research experience (Experience; 26.1%); improved their knowledge and understanding of the theory (Comprehension; 22.7%); and increased their perceived employability and awareness of career options (Careers; 20.5%). Students also commented that such projects made the subject more interesting and memorable, improved their engagement, and encouraged them to try harder (Engaged; 10.2%); improved their practical abilities (Skills; 10.2%); and improved their confidence in those abilities (Confidence; 6.8%). Finally, there were some remarks upon the cost‐effectiveness for research budgets to have student volunteers collecting data (Cost‐Effective; 3.4%).

## 4 DISCUSSION

This study aimed to examine whether involvement with RLRPs can generate reliable scientific data and function as a motivational tool for engaging tertiary science students. Results indicated that error rate in student‐collected data was minimal; dogfish were correctly sexed on 98% of occasions and 79% of measurements were within ±1 cm of ground‐truthed records. However, differences existed between Control and Experimental Groups, as well as by degree type and measurement site. Interestingly, students were more accurate where the project topic aligned specifically with their degree subject. In terms of student perceptions, surveyed students reported that they found the idea of participating in RLRP motivating, thought it was an important component of their education, and placed strong value on such involvement for future employability. Yet, students who had not participated in a RLRP felt less confident about doing so. Overall, RLRPs are well‐perceived by students, and there is strong potential for students to contribute quality scientific data to such projects.

### 4.1 Student‐based data collection

Staff attitudes toward engaging students in research has the potential to limit research‐based curricula (Brew, 2013). Few academics utilize undergraduate student involvement in RLRPs, possibly as a result of concerns regarding students' ability to collect reliable data and limited perceptions of how students develop research capability (Hattie & Marsh, 1996; Wilson et al., 2012). However, this study revealed an overall high degree of accuracy for student‐collected data. Therefore, involving students in RLRP could be advantageous to staff, particularly with regard to time efficiency through providing additional opportunities for research and a higher number of “person hours” for data collection. This also adds an incentive to the development of research‐based learning where students are encouraged and facilitated to undertake research and inquiry (Healey & Jenkins, 2009).

It is worth noting, however, that accuracy varied according to several factors. The error rate was significantly different by Treatment Group; but both the Control and the Experimental Groups were equally erroneous. However, when considered by degree stream, Marine Biology students averaged a zero error rate. Given the marine‐theme of the task, it may be that those students held greater interest or placed greater value on this activity than Biology students (who include those intending to specialize in topics such as botany, genetics, and microbiology, as well as zoology). Furthermore, some measurements appeared more difficult to collect than others, with dogfish mouth and total body length having particularly large error rates. The former is tricky to measure, while the latter required maths skills due to rulers of insufficient length. There was also variation among students, with some pairs having median error rates closer to zero than others; however, this difference was not statistically significant.

In summary, student‐collected data are reliable. However, it is essential to give students clear instructions and may be beneficial if the RLRP is a topic of direct interest or relevance to their specialist areas.

### 4.2 Student perceptions of “real life” research projects (RLRPs)

If they are to engage in research‐based learning, students holding positive attitudes toward research is critically important (Brew, 2013). In this study, students were extremely positive about practicals in general and this class in particular, and considered practicals valuable to learning. However, students reported low confidence in their own abilities. This may reflect their status as first‐year undergraduates, as well as the difference between “doing” a practical and actually “learning” from it (Abrahams & Millar, 2008).

Irrespective of Treatment Group, all students were positive regarding the benefits of RLRP and thought it was important for students to be involved with such projects. This demonstrates that all students were aware of the potential benefits of participating in RLRPs, even without being involved in one. Thus, it was not the case that by being involved in a RLRP students were biased toward perceiving value. Rather, nearly all students perceived participating in RLRPs to provide relevant experience, improve theoretical comprehension, and enhance employability. This mirrors other research regarding involvement in extracurricular activities, which employers value in employee selection and graduates value for ongoing professional and personal development (Stuart et al., 2011).

While both Treatment Groups found the idea of participating in RLRPs motivating, students who had participated in this study's RLRP reported stronger motivation and greater confidence in their abilities than their peers. Students who had participated in this study's RLRP also reported greater confidence in participating in such projects. Despite the general consensus that RLRPs are beneficial to both current learning and future employability, this means that students may not have the confidence to engage with RLRPs independently. It may be that exposing students to RLRPs within a classroom environment will grow their confidence and encourage them to seek additional opportunities in the future, thus facilitating enhanced learning and career development.

### 4.3 Study impact

This study supports the importance of the research‐teaching nexus (Brew & Boud, 1995; Harland, 2016; Healey et al., 2010; Jenkins et al., 1998, 2003; Neumann, 1994; Tight, 2016). Strengthening the links between research and teaching is important because universities have a responsibility to prepare students for professional life and research‐based learning curricula provide countless opportunities for students to develop key, discipline‐specific skills (Brew, 2013). Thus, there is a need to develop knowledge‐building communities within universities and shift students from traditional roles as consumers of knowledge into active producers of knowledge (Brew, 2006; Neary, 2010). This can be achieved by creating research partnerships between academics and students; for example, academic‐directed inquiry allowing students to engage in both acquiring existing and creating new knowledge (Levy & Petrulis, 2012). Such partnerships also enable closer contact with more intangible aspects of learning (e.g., critical approaches to knowledge, positive attitudes to learning, fostering curiosity and enjoyment; Neumann, 1994).

Involvement in RLRPs improves student motivation, provides the opportunity for responsibility and agency, and fosters collaboration between students and academics. By increasing student engagement through involvement in RLRPs, there is a justification for incorporating such involvement in curriculum development. “Educators have the privilege to shape curricula, and thereby create their students' motivational context” (Kickert et al., 2022). Additionally, given that data collected by students has a high degree of accuracy, these findings should encourage academics to actively involve undergraduate students in their research.

Practical classes have previously demonstrated success in increasing engagement among undergraduate science students (Charney et al., 2007). However, there is the potential to accentuate these benefits using RLRPs as authentic learning environments (Breen & Lindsay, 1999; Charney et al., 2007). This study built upon an existing lesson plan by adding RLRP components to a first‐year undergraduate practical class. Although these components were relatively basic in terms of broader scientific skills, framing in the context of a RLRP added value to routine tasks that could otherwise be misinterpreted as “meaningless” by students (Abrahams & Reiss, 2012; Tuan et al., 2005). Furthermore, rather than research experiences being reserved for final‐year students or those fortunate enough (or confident enough) to participate in dedicated extracurricular programs, it is important to integrate research‐based learning experiences at earlier stages so that student capacities can be progressive developed throughout their degrees (Brew, 2013; Clark & Hordosy, 2019; Howell, 2021). Accentuating standard first‐year practical classes through inclusion of a RLRP component has the potential to capture a larger number of students, overcome background characteristics that limit participation in individual research experiences, build student confidence, and create a solid foundation of research skills right from the start of the degree that can be built upon over time.

From a logistical standpoint, these additions to the lesson plan contributed approximately an extra 15 min to a 2.5‐h class. Despite a relatively small time‐investment, this resulted in higher levels of student engagement. It would therefore be feasible to consider such additions across the broader curriculum. Furthermore, these additions actually offer a time‐saving measure to academics. Many staff run practical classes directly related to their area of research, yet relatively few of these are linked with active research projects. Given the accuracy of student‐collected data revealed in this study, it is recommended that academics develop teaching materials in closer alignment with their own research objectives. This would benefit staff in terms of additional resources for data collection and subsequent increases in academic output. For instance, by collecting dogfish morphological measurements over several years, the lead author anticipates being able to investigate changes in physical characteristics and growth rates linked to sex, age, geography, fishing activity, and climate change. Thus, by aligning research and teaching, there is a heightened potential for discipline‐specific staff outputs. Students would also benefit from a dynamic curriculum that reflects current research needs, provides training in applied skills, and enhances student success.

In the long term, incorporating student involvement in RLRPs throughout the undergraduate science curriculum has the potential to positively influence course satisfaction ratings, student employability, and staff research outputs (Breen & Lindsay, 1999; Dunlop et al., 2019). We recommend academics examine their own research activities to identify what tasks exist that would align with undergraduate skills, and consider whether such tasks could be incorporated into teaching activities to offer hands‐on experience while also generating large‐scale and long‐term data sets.

### 4.4 Limitations and future work

The key limitation of this study is that it was restricted to a relatively small sample size from a single institution. It is therefore best viewed as a foundation for future research, which will broaden the impact of this work to allow application at a national or international level. In the first instance, it would be useful to reconfigure the present study in a traditional experimental format (i.e., equal number of control and experimental groups), with extended data on student demographics and background characteristics, and repeat this with multiple cohorts undertaking the same practical class over a number of years to create a larger and broader dataset. In a wider context, it would be interesting to replicate the study at different institutions, degrees, and class types. Longer term, it would be beneficial to understand the longevity of benefits arising from involvement in RLRPs, as well as staff perceptions.

It is also worth considering that there are many factors that contribute toward student engagement (Braxton, 2006; Kuh et al., 2007; Zepke & Leach, 2010). These can be intrinsic, such as student gender, race, ethnicity, and socio‐economic status; or they may be extrinsic and relate to aspects of the teacher, classroom, or institution. Many of these do not exist singularly, but instead interact intersectionally to enhance engagement or trigger disengagement. Some of these factors were controlled within this study; for example, the class was taught by the same person, in the same room, within the same institution. Some aspects even contributed to the study and were statistically assessed (e.g., degree type). However, inevitably, there were still a variety of measures beyond those considered here that could have influenced student engagement. Thus, this project provides a piece of the overall student engagement puzzle, which can be built upon in future studies.

## 5 CONCLUSION

This research demonstrates that students can collect accurate data for scientific research. In particular, student accuracy was greatest when the task aligned with their degree topic. This offers an opportunity to academics looking to build long‐term research projects requiring high people hours for measurement‐based tasks.

All surveyed students enjoyed this practical task, felt motivated by the idea of working on RLRPs, were keen to gain relevant work experience, and valued RLRPs in terms of enhancing their own comprehension and future employability. However, students who had participated in the RLRP component of this study showed greater motivation and confidence than their peers. Interestingly, unless students had participated in a RLRP, they did not feel confident about engaging with such projects. Thus, it is recommended that RLRPs are embedded early into the tertiary curricula to develop student confidence in contributing to “real life” research and encourage future engagement with such professional development opportunities.

In conclusion, incorporating RLRPs into the curriculum of undergraduate science students can have considerable benefits for both students and academic staff. For greatest success, we recommend that such projects are carefully designed, clearly implemented, and directly overlap with both student interests and staff expertise. Therefore, we invite academics to review their own research practices to identify how they can align more closely with teaching activities and student learning opportunities.

## AUTHOR CONTRIBUTIONS

**Sarah A. Marley:** Conceptualization (lead); data curation (lead); formal analysis (lead); investigation (lead); methodology (lead); software (lead); visualization (lead); writing – original draft (lead); writing – review and editing (equal). **Alessandro Siani:** Conceptualization (supporting); methodology (supporting); writing – review and editing (equal). **Stuart Sims:** Conceptualization (supporting); methodology (supporting); supervision (lead); writing – review and editing (equal).

## CONFLICT OF INTEREST

The authors declare no competing financial or nonfinancial interests that are directly or indirectly related to this work.

### OPEN RESEARCH BADGES

This article has earned Open Data and Open Materials badges. Data and materials are available at http://10.6084/m9.figshare.20361861.

## Data Availability

The data used in this project are freely available from: http://10.6084/m9.figshare.20361861
